# dbcan-nf: a Nextflow pipeline for accurate CAZyme annotation in metagenomes

**DOI:** 10.1093/bioadv/vbag304

**Published:** 2026-10-07

**Authors:** Xinpeng Zhang, Haidong Yi, Jinfang Zheng, Yanbin Yin

**Affiliations:** Department of Food Science and Technology, Nebraska Food for Health Center, University of Nebraska-Lincoln, Lincoln, NE 68588, United States; Center for Applied Bioinformatics, St. Jude Children’s Research Hospital, Memphis, TN 38105, United States; Chongqing Institute of Intelligent Medicine (CIIM), Chongqing, 400042, China; Department of Food Science and Technology, Nebraska Food for Health Center, University of Nebraska-Lincoln, Lincoln, NE 68588, United States

## Abstract

**Motivation:**

Carbohydrate-active enzymes (CAZymes) are crucial for carbohydrate degradation in any ecological environment. However, CAZyme annotation is not easy in whole-genome shotgun (WGS) metagenomes, due to the lack of accurate, scalable, robust, and reproducible workflows. We previously published dbCAN as a web server and associated standalone tool run_dbcan that performs CAZyme annotation with assembled genomes as the input but not with raw WGS reads.

**Results:**

Here, we present dbcan-nf, an accurate and scalable Nextflow pipeline integrating run_dbcan as a component step, for automated CAZyme annotation in metagenomes. dbcan-nf supports multiple analytical routes, including individual sample assembly (IA), multisample coassembly (CA), assembly-free (AF), and subsampling (SS), and accepts both short and long reads. Using five benchmark datasets, we showed that dbcan-nf (IA and AF routes) had a much higher accuracy (recall and F1 score) and detected more CAZyme families than the competing tool Cayman. Among the different routes, dbcan-nf-IA produced the most accurate CAZyme annotation, especially when the sequencing depth is sufficient (e.g., >1.0 Gb). However, as a read assembly-based method, dbcan-nf-IA had a higher computational cost than Cayman. Finally, dbcan-nf leverages Nextflow-supported high-performance computing execution for workflow-level scalability through concurrent processing of multiple samples.

**Availability and implementation:**

https://github.com/bcb-unl/dbcan-nf.

## 1 Introduction

Metagenomics has greatly enhanced our understanding of complex microbiomes in various environments, such as the human gut (Almeida *et al.* 2021). Central to microbial community function is the metabolic potential to degrade and utilize diverse carbohydrates, primarily mediated by carbohydrate-active enzymes (CAZymes) (Kaoutari *et al.* 2013; Wardman *et al.* 2022). CAZymes include glycoside hydrolases (GHs), polysaccharide lyases (PLs), carbohydrate esterases (CEs), glycosyltransferases (GTs), carbohydrate-binding modules (CBMs), and auxiliary activity (AA) enzymes (Drula *et al.* 2022), all of which are fundamental to global carbon cycling, microbe–host interactions, and ecosystem stability (Huang *et al.* 2018; Zheng *et al.* 2023b).

We developed dbCAN as a web server that takes assembled genomes/contigs for automated CAZyme annotation, and run_dbcan as a command-line version of dbCAN (Yin *et al.* 2012; Zhang *et al.* 2018; Zheng *et al.* 2023a). Other tools for CAZyme annotation, such as CUPP (Barrett *et al.* 2023) and CAZyLingua (Thurimella *et al.* 2025), require protein sequences as input. However, using raw reads from whole-genome shotgun (WGS) metagenomic samples for CAZyme (occurrence and abundance) profiling has been challenging. Cayman was recently published to directly take metagenomic reads as the input for CAZyme profiling (Ducarmon *et al.* 2026). Cayman maps WGS reads to a precomputed reference gene catalog from the global microbial gene catalog (GMGC) (Coelho *et al.* 2022). However, published in 2022, the GMGC contains human gut metagenomes mainly from the USA, Europe, and China, lacking metagenomes from non-industrialized populations (Abdill *et al.* 2022) [mostly became available after 2022, e.g. (Carter *et al.* 2023)]. Cayman’s reference gene catalog, therefore, is biased toward industrialized human microbiomes, and importantly lacks representations from understudied microbiomes (e.g. those from plant-associated environments, non-marine waters, soils, most animals, and non-industrialized human populations). Consequently, Cayman’s reference-based CAZyme profiling will likely underestimate the CAZyme diversity in understudied microbiome samples. Moreover, there are numerous WGS data analysis tools, each with unique dependencies and parameter settings. This poses significant difficulties for researchers to profile CAZyme abundances in microbiomes reproducibly and reliably. To standardize CAZyme annotation in WGS data, we recently published a step-by-step workflow or protocol as BioRxiv preprint (Zheng *et al.* 2024). This protocol describes a collection of Bash one-liners and Python scripts that have to run one by one in a manual fashion.

The nf-core framework (Ewels *et al.* 2020) provides an ideal solution to develop fully automated computational workflows by leveraging the Nextflow framework (Di Tommaso *et al.* 2017). It ensures pipeline portability, scalability, reproducibility, and seamless integration with high-performance computing (HPC) environments. Nextflow also enables workflow robustness by allowing interrupted runs to be resumed through Nextflow caching. Building upon this robust infrastructure, we developed dbcan-nf, a scalable Nextflow pipeline to implement our recently developed protocol (Zheng *et al.* 2024) for fully automated and more accurate CAZyme annotation in WGS microbiome samples. Such a CAZyme annotation Nextflow pipeline is urgently needed by the microbiome and CAZyme research communities. The scalability of dbcan-nf is enabled by Nextflow’s ability for job parallelism on the HPC system, supporting the concurrent processing of increasing numbers of independent samples through task distribution on HPC nodes.

Note dbcan-nf is not a new algorithm or method for CAZyme annotation. Instead, it is a new Nextflow pipeline that implements four different routes [individual sample assembly (IA), coassembly (CA), assembly-free (AF), and subsampling (SS)] to automate CAZyme annotation with a single or multiple samples of WGS reads as the input. This paper describes the overall design of the four different routes. More importantly, it presents a comprehensive evaluation of the four routes of dbcan-nf and a comparison against Cayman in terms of annotation accuracy at the CAZyme family level and computational efficiency.

## 2 Methods

### 2.1 Installation and database files

The dbcan-nf pipeline (Fig. 1) is developed following the nf-core community standards (Ewels *et al.* 2020; Langer *et al.* 2025) and requires Nextflow as a fundamental prerequisite. Users can choose Docker, Apptainer/Singularity, or Anaconda to manage the software environment, ensuring high reproducibility and portability across different computing infrastructures. All necessary databases for WGS read processing and CAZyme annotation, such as CAZyDB (Drula *et al.* 2022), dbCAN HMMdb (Yin *et al.* 2012), and dbCAN-sub HMMdb (Zheng *et al.* 2023a), should be predownloaded separately. This separation of dbcan-nf installation and database preparation significantly reduces initial runtime, especially for resource-intensive databases like Kraken2 (Wood *et al.* 2019).

**Figure 1:**
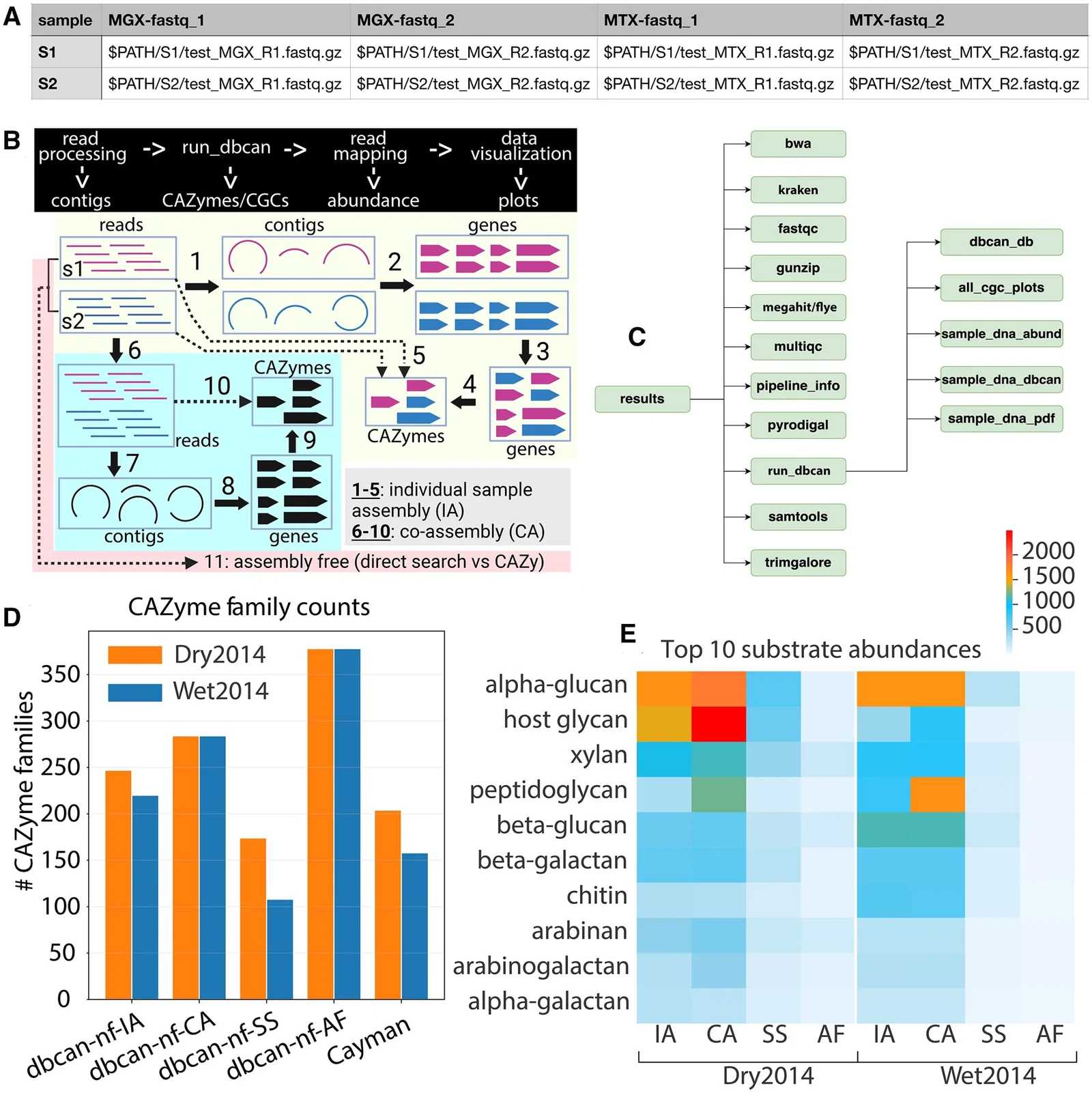
Overview of the dbcan-nf workflow and performance evaluation. (A) An example input file (tsv format) including two samples, each with a pair of MGX and MTX FASTQ file paths in the user’s computer file system. (B) The workflow consists of four modules, as shown at the top of the figure. Three analysis routes are illustrated: individual sample assembly (IA), coassembly (CA), and assembly-free (AF). Reads from two example metagenomic samples (s1 and s2) are processed through these routes for CAZyme annotation. The workflow contains four modules as shown at the top in black background. Three routes are shown with different background colors: light yellow for individual sample assembly (IA), light blue for coassembly (CA), and light red for assembly-free (AF). Reads of two example metagenomic samples (s1 and s2) are processed with the three routes for CAZyme annotation. The IA route involves read assembly of each individual sample (Steps 1–5), while the CA route requires pooling the reads first and coassemble the pooled reads (Steps 6–10). Once CAZymes and CGCs are identified by run_dbcan (Step 4 for IA and Step 9 for CA), reads are mapped back (dashed lines, 5 and 10) to calculate their abundances in different samples. The third route is AF (Step 11), which directly searches reads against the CAZy preannotated CAZyme protein sequences. The IA and CA routes can be slightly modified by subsampling a subset of reads instead of using all reads for assembly, which is also implemented in dbcan-nf. (C) Result folder organization output from dbcan-nf. Each program called in the workflow generates a separate folder with intermediate files for easy access and review. The run_dbcan folder contains subfolders with final tabular files and PDF visualization plots for CAZyme abundances in different samples. (D) An example plot as one of the output files from dbcan-nf for CAZyme family counts. Two SRA read files (ERR7745896 for Wet2014 and ERR7738162 for Dry2014) are used here from fecal samples of Hadza hunter-gatherers (Carter *et al*. 2023). Results of the four routes (IA, CA, SS, and AF) were direct outputs of dbcan-nf, while the result from Cayman was added afterwards for comparison. (E) An example plot as one of the output files from dbcan-nf for glycan substrate abundances. The heatmap shows the summed abundance of CAZymes targeting different glycan substrates in the two SRA read samples (ERR7745896 for Wet2014 and ERR7738162 for Dry2014).

Resource requirements vary depending on input data size and sequencing depth. In our benchmarking tests, we allocated 32 CPUs and 200 GB of RAM, providing a reference for the typical computational resources required to efficiently execute the pipeline.

### 2.2 Input files

The dbcan-nf pipeline requires a comma-separated sample sheet as the input file (Fig. 1A), enabling the simultaneous processing of multiple samples within a single analysis. Each sample entry in the sheet must specify: (i) a unique prefix (e.g. S1 and S2) to distinguish samples, (ii) paths to metagenomic (MGX) FASTQ read files (compressed files supported), and optionally, and (iii) paths to metatranscriptomic (MTX) FASTQ read files if available. Currently, dbcan-nf supports both Illumina paired-end short reads and PacBio/Nanopore long reads. Example input files and detailed instructions are provided on dbcan-nf’s GitHub repository page.

Although the dbcan-nf pipeline is designed to process FASTQ reads for CAZyme annotation, users have the option to bypass the read processing steps and directly input FASTA sequences of assembled contigs or proteins. For that, the dbcan-nf pipeline will essentially only run the run_dbcan step.

### 2.3 Workflow design

The dbcan-nf workflow consists of four major steps: reads processing, run_dbcan CAZyme annotation, read mapping, and data visualization (Fig. 1B). When users input multiple MGX read samples, two major routes are available to choose from: IA and CA. For individual sample assembly, users provide multiple samples of reads as the input, and each sample is processed individually (Fig. 1B, Steps 1–5). Specifically, raw reads of each sample first undergo preprocessing with FASTQC (https://www.bioinformatics.babraham.ac.uk/projects/fastqc/) and Trim Galore (Capella-Gutiérrez *et al.* 2009) for adapter trimming and quality filtering. Contamination removal using Kraken2 and KrakenTools (Lu *et al.* 2022) is provided as an optional step. For read assembly into contigs (nucleotide FASTA format, FNA file), MEGAHIT (Li *et al.* 2015) is utilized for short reads, while Flye (Kolmogorov *et al.* 2020) is employed for long reads. Protein-coding sequences are predicted from contigs using Pyrodigal (Larralde 2022), generating output files of amino acid sequences (amino acid FASTA format, FAA file) and gene feature annotations (general feature format, GFF file). Users have the option to choose whether they want to combine protein sequences from multiple samples followed by sequence redundancy removal. Subsequently, the FAA and GFF files of all samples are used as the input to the run_dbcan tool (Zheng *et al.* 2023a, 2023b) for CAZymes and CAZyme gene clusters (CGCs). To quantify CAZyme and CGC abundances, the original MGX reads (or optional MTX reads) are mapped back to the contig FNA file using BWA-MEM (short reads) or Minimap2 (long reads) (Li 2013, 2018) using default parameters. Mapped reads are filtered with the mapping quality score >30 and the alignment sequence identity >98%. Finally, the abundance [as the Reads Per Kilobase Million (RPKM) or Transcripts Per Million (TPM) values] calculation and visualization outputs are generated using built-in Python scripts within the workflow.

For coassembly of multiple samples (usually from the same study design) (Fig. 1B, Steps 6–10), reads from multiple samples are pooled together (Step 6) for a coassembly into contigs (Step 7). The gene prediction (Step 8), CAZyme annotation (Step 9), and read mapping back (Step 10) are conducted in the same way as the individual sample assembly route. Additionally, an assembly-free route (Fig. 1B, Step 11) is provided to facilitate read mapping-based analyses using DIAMOND blastx (Buchfink *et al.* 2021) search against protein sequences of CAZymes from the CAZyDB (Drula *et al.* 2022).

Finally, dbcan-nf also includes a subsampling option, which uses Seqkit (Shen *et al.* 2016) to randomly extract a representative subset of reads from each sample or pooled samples. This is designed to reduce computational demands and enable rapid preliminary CAZyme analyses. Overall, this comprehensive workflow is adapted from our previously established dbCAN protocol (Zheng *et al.* 2024).

### 2.4 Output files

The dbcan-nf pipeline will generate nested folders (Fig. 1C) as the results. Two types of output files, plain text files and visualization files, are organized in these folders. The plain text files include TSV files with occurrences and abundances of CAZymes, CGCs, and glycan substrates in each input sample. The visualization files include PDF files with barplots and heatmaps for occurrences and abundances for CAZyme families (Fig. 1D) and glycan substrates (Fig. 1E). If specific CGCs are selected by users, plots are also generated to visualize the per-base read mapping on the selected CGCs across different samples. Note that CGC profiling does not work for the assembly-free route, as CGCs are predicted in assembled contigs.

### 2.5 Benchmark datasets and performance evaluation

We constructed five benchmark datasets (Table 1) to quantitatively evaluate the performance of different routes of dbcan-nf and compare them with the competing tools such as Cayman. These datasets were used for different evaluation purposes, depending on how they were generated, the availability of ground-truth CAZyme annotation, the sequencing depths, and their sample sizes. Detailed descriptions of these datasets (Table 1, available as supplementary data at *Bioinformatics Advances* online) and competing tools (Table 2, available as supplementary data at *Bioinformatics Advances* online) are provided in the Supplementary Methods, available as supplementary data at *Bioinformatics Advances* online.

**Table 1:** Summary of five benchmark datasets.

Benchmark dataset	Description	No. of samples	Evaluation purposes	Reference
CAZy-rand-10	Illumina short reads simulated from 10 RefSeq genomes annotated by CAZy	10	CAZyme annotation accuracy, route selection	Drula *et al.* (2022)
Mock-stag-24	Illumina short reads sequenced from 24 reference genomes from human and mouse guts	18	CAZyme annotation accuracy, sequencing depth impact, route selection	Treichel *et al.* (2026)
Mock-stag-70	Nanopore long reads sequenced from 70 reference genomes from human and mouse guts	4	CAZyme annotation accuracy, long read input, scalability	Treichel *et al.* (2026)
Hadza-gut	Illumina short reads from fecal microbiome samples of Hadza hunter-gatherers in Africa	2	Route selection, understudied microbiomes	Carter *et al.* (2023)
Wastewater	Illumina short reads from wastewater samples from India	20	Computational cost, scalability, understudied microbiomes	Singh *et al.* (2026)

Briefly, three benchmark datasets (*CAZy-rand-10*, *Mock-stag-24*, and *Mock-stag-70*) have reference genomes (Tables 3–5, available as supplementary data at *Bioinformatics Advances* online). Their genome-based CAZyme annotations were used as the ground-truth data to compare the CAZyme annotation accuracy between different tools and dbcan-nf route selections (Tables 6–8, available as supplementary data at *Bioinformatics Advances* online). Once their simulated reads (*CAZy-rand-10*) or sequenced reads (*Mock-stag-24* and *Mock-stag-70*) were processed by different tools (Table 2, available as supplementary data at *Bioinformatics Advances* online) or routes (Fig. 1B), true positives (TP) were defined as CAZyme families present in both predictions and ground-truths. False positives (FP) were defined as predicted CAZyme families absent from ground-truths. False negatives (FN) were defined as CAZyme families in ground-truths but absent in predictions. Precision, recall, and F1 score were calculated at the CAZyme family level:


$$\mathrm{Recall}=\;\frac{\mathrm{TP}}{\mathrm{TP}+\mathrm{FN}}$$

$$\mathrm{Precision}=\;\frac{\mathrm{TP}}{\mathrm{TP}+\mathrm{FP}}$$

$$F1=2\times \frac{\mathrm{Precision}\times \mathrm{Recall}}{\mathrm{Precision}+\mathrm{Recall}}$$

For the two real-world metagenomic datasets: *Hadza-gut* and *Wastewater*, we do not have reference genomes and their CAZymes as the ground-truth data. Therefore, the evaluation of CAZyme annotation performance (i.e. recall, precision, and F1 score values) was not possible. *Hadza-gut* and *Wastewater* were mainly used to evaluate the scalability and route selection of dbcan-nf, and to compare dbcan-nf against Cayman in an understudied non-gut microbiome context (Table 1, Tables 9–11, available as supplementary data at *Bioinformatics Advances* online), including the comparison of computing time (Wall-clock time and total CPU time) and peak memory use. These analyses were used to compare the detected CAZyme repertoire breadth and computational requirements rather than annotation accuracy.

Specifically, Cayman was run with the habitat-specific reference gene catalogs (Table 12, available as supplementary data at *Bioinformatics Advances* online), e.g. the human gut reference gene catalog for *CAZy-rand-10* and *Hadza-gut*, the combined human and mouse gut reference gene catalog for *Mock-stag-24* and *Mock-stag-70*, and the wastewater reference gene catalog for *Wastewater*. This allowed us to evaluate Cayman under catalog settings matched to the corresponding microbiome context.

For microbiomes from wastewater, freshwater, and soil, Cayman has small, precomputed reference gene catalogs from GMGC (Coelho *et al.* 2022). Some of the most studied microbiomes from animal hosts (e.g. cow rumens) and ecological environments (e.g. plants and hydrothermal vents) are excluded from Cayman’s gene catalogs. The human gut microbiome in Cayman also lacks representation from understudied human populations (e.g. Africans). Therefore, the *Hadza-gut* and *Wastewater* benchmark datasets were selected to run dbcan-nf and Cayman to compare their annotated CAZyme families and glycan substrate profiles (Tables 9 and 10, available as supplementary data at *Bioinformatics Advances* online).

## 3 Results

### 3.1 dbcan-nf has a higher CAZyme annotation accuracy than Cayman

We built three benchmark datasets (*CAZy-rand-10*, *Mock-stag-24*, and *Mock-stag-70*) (Table 1) to evaluate the CAZyme annotation accuracy of dbcan-nf and compare against competing tools (see details in the Supplementary Methods, available as supplementary data at *Bioinformatics Advances* online).

The competing tools include Cayman (Ducarmon *et al.* 2026), CUPP (Barrett *et al.* 2023), and CAZyLingua (Thurimella *et al.* 2025) (Table 2, Table 2, available as supplementary data at *Bioinformatics Advances* online). WGS reads in FASTQ format were used directly as input (Table 2) to dbcan-nf and Cayman. For *CAZy-rand-10* and *Mock-stag-24*, dbcan-nf was run using the individual sample assembly (dbcan-nf-IA) route and the assembly-free route (dbcan-nf-AF). Cayman uses an older version of dbCAN HMMdb (v9) for building their reference CAZyme gene catalogs, while dbcan-nf uses the latest version of dbCAN HMMdb (v14). For a fair comparison, we also downgraded dbcan-nf using the same dbCAN HMMdb v9 as Cayman (dbcan-nf-IA-v9 and dbcan-nf-CA-v9 in Table 3).

**Table 2:** Feature comparison of different CAZyme annotation tools.

Tool	dbcan-nf	Cayman	CUPP	CAZyLingua
Type	Nextflow workflow	Standalone annotation tool	Standalone tool with webserver	Standalone tool
Inputs	Sample sheet with links to MGX FASTQ	MGX FASTQ	Protein FASTA	ProtT5 embeddings of protein FASTA
Outputs	Presence and abundance of CAZymes (dbCAN fams and subfams), CGCs, EC, substrates, and visualization	Presence and abundance of CAZymes (CAZy fams and subfams) and substrates^a^	Presence of CAZymes (CAZy fams and subfams), CUPP groups, ECs	Binary CAZyme/non-CAZyme labels (CAZy fams and subfams)
Required databases	CAZy FASTA, dbCAN, dbCAN-sub HMMs, fam-substrate mapping	GMGC CAZyme gene FASTA, fam/subfam-substrate mapping	CUPP peptide-pattern library	CAZyme ProtT5 embeddings, trained classifiers

a No script is provided in the Cayman package for substrate abundance summarization.

**Table 3:** Comparison of the CAZyme annotation accuracy among different tools.

Dataset	CAZy-rand-10 (10 samples)			Mock-stag-24 (18 samples)		
Tool/method	Mean recall	Mean precision	Mean F1	Mean recall	Mean precision	Mean F1
Cayman	0.67	0.98	0.80	0.62	0.97	0.76
dbcan-nf-AF	0.80	0.97	0.88	**0.87**	0.90	**0.89**
dbcan-nf-IA	**0.83**	**1.00**	**0.90**	0.76	**0.98**	0.83
dbcan-nf-AF-v9	0.70	0.97	0.82	0.70	0.91	0.79
dbcan-nf-IA-v9	0.80	**1.00**	0.88	0.73	**0.98**	0.81
CAZyLingua	0.67	0.70	0.69	0.62	0.74	0.66
CUPP	0.60	0.92	0.72	0.57	0.90	0.68

The bold with underline values are the highest values in the column.

For CUPP and CAZyLingua, WGS reads of each sample were first assembled into contigs using MEGAHIT (Li *et al.* 2015). Protein-coding genes were then predicted from the assembled contigs using Pyrodigal (Larralde 2022), generating protein FASTA files for the downstream CAZyme annotation. The predicted protein sequences were then used as input for CUPP (Table 2). For CAZyLingua, protein sequences were first converted into numerical embeddings using ProtT5 (Elnaggar *et al.* 2022), and these embeddings were used as input for CAZyLingua (Table 2). run_dbcan was excluded in all comparisons, as it is a component step of dbcan-nf.

*CAZy-rand-10* has 10 samples of simulated reads from 10 reference genomes with different relative abundances (Table 3, available as supplementary data at *Bioinformatics Advances* online) and all the 10 samples have the same depth and size (1.67 million 2 × 150-bp paired-end reads, 0.50 Gbp, Table 6, available as supplementary data at *Bioinformatics Advances* online). Accordingly, this benchmark considers the microbiome compositional variations (Table 3, available as supplementary data at *Bioinformatics Advances* online) under a controlled sequencing depth. On these 10 simulated read samples, dbcan-nf-IA had the highest mean recall, precision, and F1 score (Table 3, Fig. 2A), followed by dbcan-nf-IA-v9 as the second-best method. dbcan-nf-AF and dbcan-nf-AF-v9 also had better mean recall and F1 score than Cayman, although they are all read mapping-based methods. This is likely because dbcan-nf-AF uses the more sensitive DIAMOND blastx search against all CAZy-annotated CAZyme proteins for read mapping (read alignment coverage >90% and sequence identity >80%), while Cayman uses the faster but less sensitive BWA-MEM for read mapping (alignment length >45 bp and sequence identity >97%).

**Figure 2:**
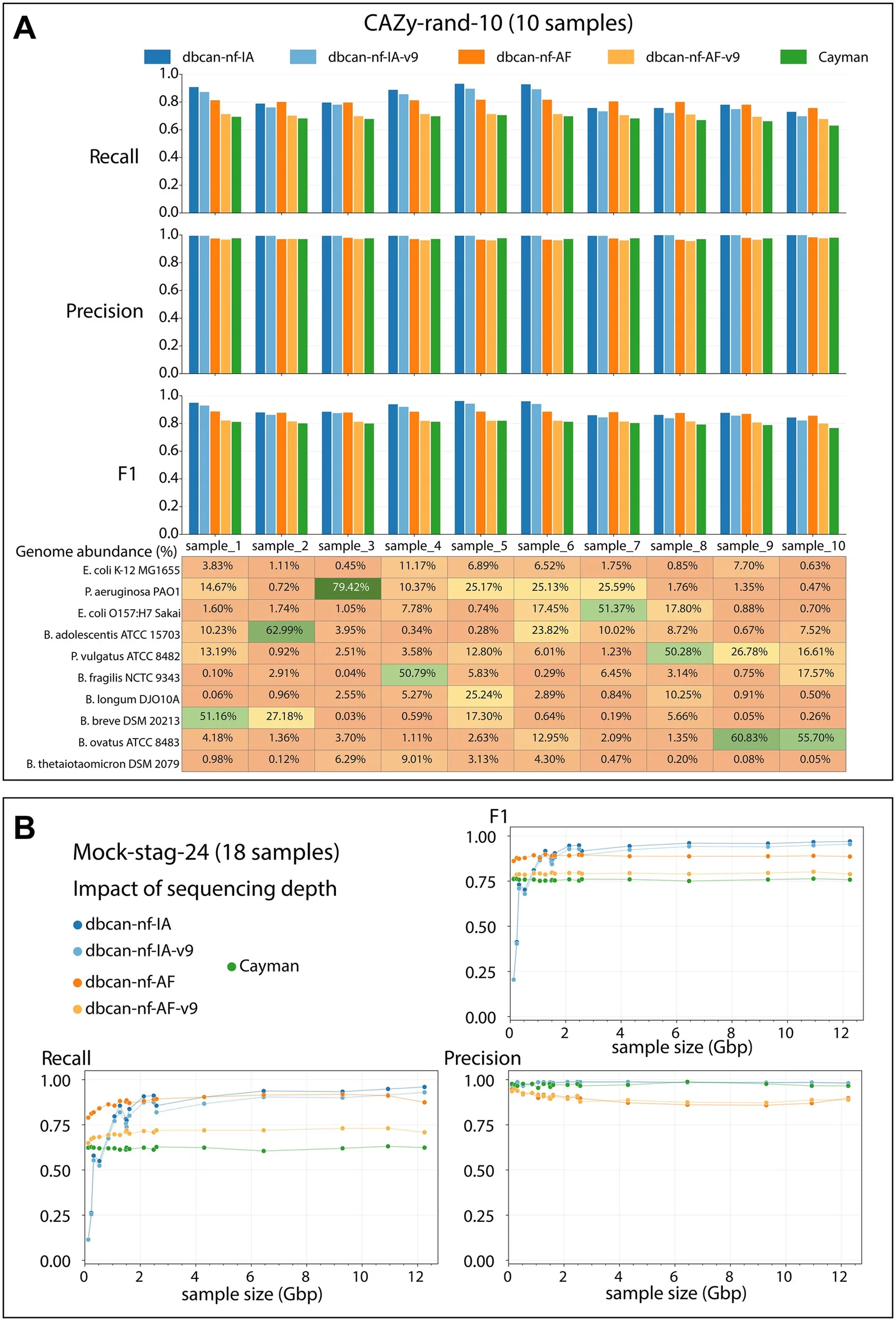
Comparison of CAZyme annotation accuracy among different routes of dbcan-nf and Cayman. (A) Barplot of recall, precision, and F1 score of 10 simulated read samples of CAZy-rand-10. The relative abundances of the 10 reference bacterial genomes are shown as a table under the barplot. The full names of the 10 bacteria and the full data are provided in Table 3, available as supplementary data at *Bioinformatics Advances* online. (B) Plots of sequencing depths (*x*-axis) versus recall, precision, and F1 score of the 18 samples of Mock-stag-24. Sample size is calculated as the total base pairs in the reads. The full data are provided in Tables 6 and 7, available as supplementary data at *Bioinformatics Advances* online.

*Mock-stag-24* is a more complex dataset including 18 samples of sequencing reads from 24 reference genomes with different relative abundances (Table 4, available as supplementary data at *Bioinformatics Advances* online). Importantly, these 18 samples have varying depths (0.4–40.6 million 2 × 150-bp paired-end reads, 0.12–12.25 Gbp, Table 7, available as supplementary data at *Bioinformatics Advances* online). Unlike *CAZy-rand-10*, reads in *Mock-stag-24* were not computationally simulated from reference genomes. Instead, DNAs were individually extracted from 24 gut bacterial isolates and mixed in a staggered distribution (i.e. different relative abundances of different bacteria; Table 4, available as supplementary data at *Bioinformatics Advances* online) (Treichel *et al.* 2026). The 18 read samples were sequenced at two different sequencing facilities from the mixed DNAs with increasing depths (Table 7, available as supplementary data at *Bioinformatics Advances* online) and the relative abundance of different bacteria differs among samples (Table 4, available as supplementary data at *Bioinformatics Advances* online). Compared to *CAZy-rand-10*, this *Mock-stag-24* benchmark considers not only the microbiome compositional variations (Table 4, available as supplementary data at *Bioinformatics Advances* online) but also the varying sequencing depth (Fig. 2B). Due to this sample complexity, the performance (especially recall) of most tools dropped in *Mock-stag-24* compared to *CAZy-rand-10* (Table 3). Figure 2B explicitly resolves the influence of sequencing depth to the observed between-sample variation.

Overall, dbcan-nf-IA, dbcan-nf-IA-v9 and Cayman all had very high precision (>0.97) on the two benchmark datasets (Fig. 2), suggesting a very low false discovery rate (FDR) in their predictions. Notably, different routes of dbcan-nf had much higher recall and F1 score values (i.e. better CAZyme annotation accuracy) than Cayman, even when the same dbCAN HMMdb v9 was used (Fig. 2). CUPP and CAZyLingua had much lower F1 scores than dbcan-nf and Cayman. Given their lower performance and the fact that they do not take WGS reads as input, CUPP and CAZyLingua were excluded from all subsequent comparisons.

### 3.2 dbcan-nf-IA is the most accurate method when the sequencing depth is >1 Gbp

Since the 18 *Mock-stag-24* samples have a staggered distribution of their sequencing depths, we plotted the sequencing depths (sample sizes in gigabase pairs) against the recall, precision, and F1 score values of the 18 samples (Fig. 2B). We observed that dbcan-nf-IA began to surpass dbcan-nf-AF when the sequencing depth reached 2.13 Gbp for recall and 1.07 Gbp for F1 score (Fig. 2B), while the precision was consistently higher in dbcan-nf-IA than dbcan-nf-AF. The recall was below 0.6 for dbcan-nf-IA when the sequencing depths were below 0.52 Gbp (Table 7, available as supplementary data at *Bioinformatics Advances* online), which pulled down the mean recall value (Table 3, Fig. 2B). In contrast, the read mapping-based dbcan-nf-AF and Cayman were less affected than dbcan-nf-IA by the low sequencing depths (Fig. 2B). Importantly, the recall and F1 score of dbcan-nf-IA already surpassed that of Cayman at 0.85 Gbp sequencing depth, and the precisions of the two tools were very close and consistently higher than dbcan-nf-AF at all sequencing depths. Overall, dbcan-nf-IA is the most accurate method with higher F1 scores in samples with >1.07 Gbp sequencing depths (Table 7, available as supplementary data at *Bioinformatics Advances* online, Fig. 2B).

### 3.3 dbcan-nf-IA recovers more CAZyme families than Cayman in real-world metagenomic samples, has a higher computational cost, and benefits from HPC for multisample processing with satisfactory scalability

In addition to CAZyme annotation accuracy, we also included two real-world datasets: *Hadza-gut* and *Wastewater*. They were mainly used to evaluate the computational efficiency, scalability, and route selection of dbcan-nf (Table 1). Given that Cayman uses biased reference gene catalogs lacking representation from less studied microbiomes (e.g. non-industrialized human populations and non-marine waters), we also used the *Hadza-gut* and *Wastewater* datasets to compare the numbers of CAZyme families detected by Cayman and the four different routes of dbcan-nf (Tables 9–11, available as supplementary data at *Bioinformatics Advances* online) in an understudied microbiome context. Importantly, the two datasets have no reference genomes, and thus we do not have the ground-truth data to evaluate the CAZyme annotation accuracy of different tools. Because ground-truth annotations were unavailable, the additional families detected in these real-world datasets could not be classified as true or false positives. These analyses therefore compared the detected CAZyme repertoire breadth rather than the annotation accuracy.

The *Hadza-gut* dataset contains two deeply sequenced gut microbiome samples from Hadza hunter-gatherers (Table 1). The Wet2014 sample has 122 million 140 × 2 PE reads of 33.67 Gbp (Table 9, available as supplementary data at *Bioinformatics Advances* online), while the Dry2014 sample has 48 million 2 × 140-bp paired-end reads of 13.43 Gbp. For the subsampling route of dbcan-nf (dbcan-nf-SS), 20 million reads were randomly subsampled from each sample. For the coassembly route (dbcan-nf-CA), we pooled reads of the two samples together for a coassembly and reported the same number of CAZyme families (Fig. 1D), computing time, and peak memory use (Fig. 3A) for the two samples. All tools for this dataset were run on a standalone desktop Linux server with 32 CPUs and 200 GB of RAM.

**Figure 3:**
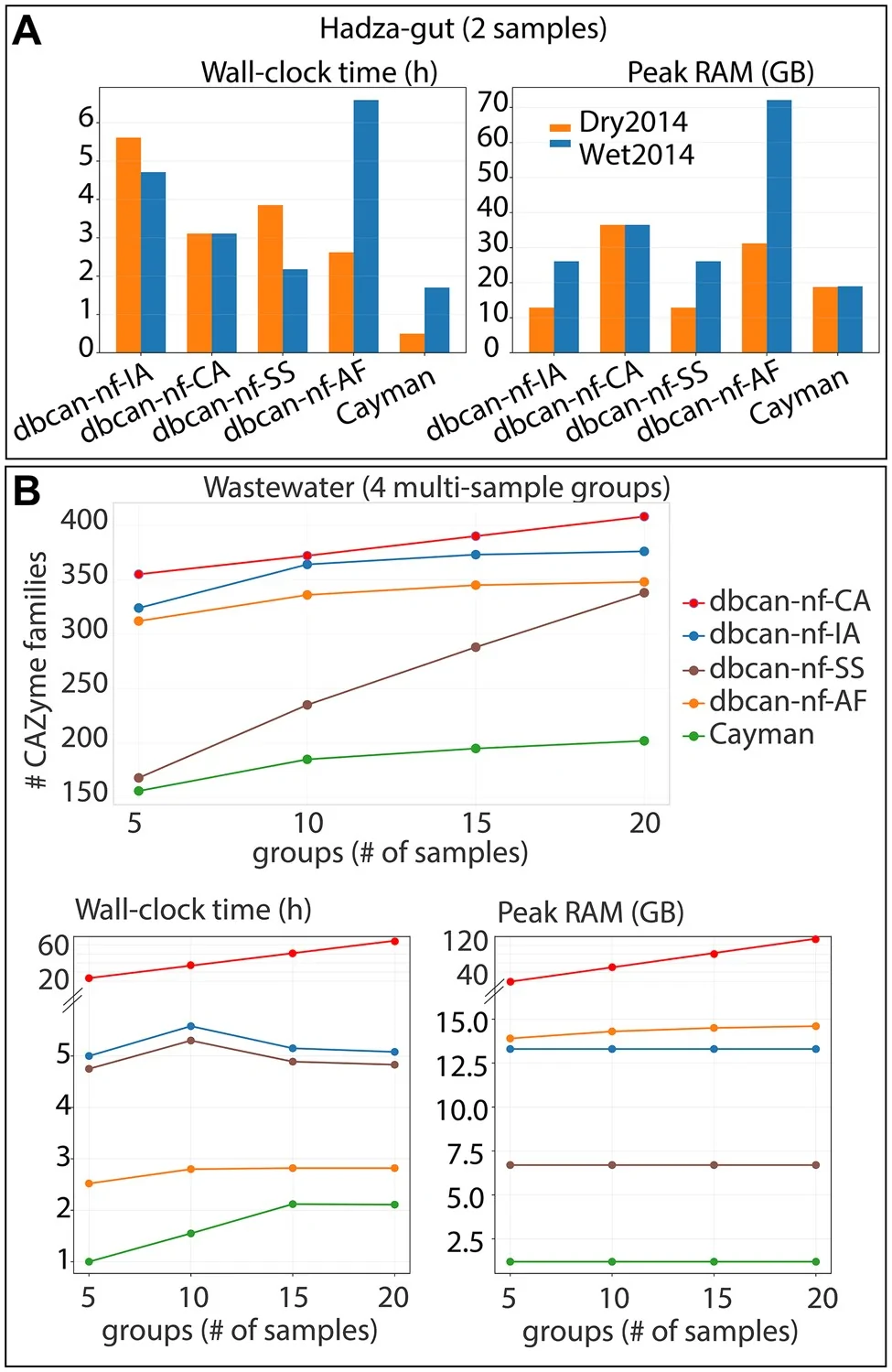
Comparison of computational costs among different routes of dbcan-nf and Cayman. (A) Barplots of computing time (wall-clock time) and peak memory use on the Hadza-gut dataset (two samples: Wet2014 and Dry2014). (B) Top: plots of # of samples in multisample groups (*x*-axis) versus total number of unique CAZyme families (*y*-axis) detected in the multisample groups. Bottom: plots of number of samples in multisample groups (*x*-axis) versus wall-clock time and peak memory use (*y*-axis) when multiple samples are processed as a group on the HPC cluster. Wall-clock time measures the elapsed time from workflow initiation to completion, and not the aggregated CPU hours on all used nodes. Peak RAM measures the highest peak RAM consumed among all used nodes, and not the sum of peak RAM used across all nodes. The experiment evaluates multisample workflow throughput and not formal strong or weak scaling of an individual sample.

We found that dbcan-nf-AF detected the highest number of CAZyme families (Fig. 1D), followed by dbcan-nf-CA and dbcan-nf-IA, in agreement with the observation made in Fig. 2 and Table 3 that dbcan-nf-AF tends to have a higher recall. Due to its biased reference CAZyme catalog and its use of dbCAN HMMdb v9, Cayman detected the lowest number of CAZyme families except for dbcan-nf-SS (Fig. 1D). Hadza gut microbiomes were predicted to have ∼730 bacterial species (Carter *et al.* 2023), much higher than industrialized gut microbiomes. The compositional complexity (species diversity and their relative abundance variation) of the *Hadza-gut* dataset is thus much higher than *CAZy-rand-10* and *Mock-stag-24*. Therefore, the amount of input reads for assembly (corresponding to sequencing depth) has a major impact on how many low-abundance genomes and their CAZymes can be detected in assembled contigs. This could be the main reason that dbcan-nf-SS detected the lowest number of CAZyme families. dbcan-nf-IA, dbcan-nf-SS, and Cayman all detected higher numbers of CAZyme families in Dry2014 (Fig. 1D), in agreement with the previous paper (Carter *et al.* 2023). All four dbcan-nf routes revealed much higher CAZyme abundances for the degradation of alpha-glucans (e.g. starch) and host glycans (e.g. mucin) in Dry2014 (Fig. 1E), likely due to higher meat and tuber consumption in the dry season by Hadza hunter-gatherers (Carter *et al.* 2023). Cayman does not provide a function in its software package for substrate abundance summarization, and therefore a comparison of substrate abundance results between Cayman and dbcan-nf was not possible.

The *Wastewater* dataset contains 20 wastewater samples from India (12–24 million 2 × 150-bp paired-end reads, 3.6–7.3 Gbp, Table 10, available as supplementary data at *Bioinformatics Advances* online). In addition to the full (20) sample group, we also formed three groups with subsets (5, 10, 15 samples per group; Table 11, available as supplementary data at *Bioinformatics Advances* online). Each sample group was used as input to the different dbcan-nf routes and Cayman to evaluate their computational resource use and workflow-level parallel processing of increasing numbers of samples. This is particularly relevant for dbcan-nf-CA, as reads from all samples in the group were pooled together for a coassembly, and only one number of CAZyme families was reported for each group. However, for other dbcan-nf routes and Cayman, each sample of a group was processed individually and the number of unique CAZyme families from all samples in the group was reported (top, Fig. 3B). For dbcan-nf-SS, 5 million reads were randomly subsampled from each sample. All tools on the four groups of samples were run on an HPC system using the Slurm workload manager. With HPC, individual samples were processed concurrently on available computing nodes. The number of nodes used during each sample group run was dynamically determined by Slurm rather than fixed in advance. We evaluated how the elapsed wall-clock time (from workflow initiation to completion) and the peak RAM use changed as the number of input samples increased from 5 to 20. This experiment assessed the multisample workflow computational cost (bottom, Fig. 3B) rather than the aggregated CPU time and RAM use among all samples in a multisample group.

We found that dbcan-nf-CA consistently detected the highest numbers of unique CAZyme families for all multisample groups, followed by dbcan-nf-IA and dbcan-nf-AF (top, Fig. 3B). Cayman detected the lowest number of CAZyme families (202 by Cayman, 376 by dbcan-nf-IA, and 408 by dbcan-nf-CA when using all 20 samples). This low detection rate is likely due to the limited representation of wastewater sequences in the underlying reference gene catalog of Cayman. As the number of samples included in each group increased from 5 to 20, all methods detected more unique CAZyme families at the cohort level. The dbcan-nf-IA and dbcan-nf-AF curves began to plateau, whereas other curves continued to increase. Because both the number and composition of reads changed across the multisample groups, this trend (top, Fig. 3B) reflects the cumulative CAZyme repertoire recovery across an expanding cohort rather than the increased sequencing depth of an individual sample.

Although Cayman recovered the fewest CAZyme families in both *Hadza-gut* and *Wastewater* datasets, it had the highest computational efficiency (Fig. 3A and bottom, Fig. 3B). This is likely because it uses a rapid read mapping tool BWA-MEM, against smaller habitat-specific reference CAZyme gene catalogs. Specifically on the *Wastewater* dataset, Cayman consistently used the lowest wall-clock time and peak RAM (∼2 h and 1.2G RAM for the 20-sample group) (bottom, Fig. 3B, Table 11, available as supplementary data at *Bioinformatics Advances* online). dbcan-nf-AF completed the 20-sample analysis in ∼2.8 h and used 14.6 GB peak RAM, whereas dbcan-nf-SS finished in ∼4.8 h and used 13.3 GB peak RAM. dbcan-nf-IA was less computationally efficient, consuming ∼5.1 h and 13.3 GB peak RAM for the 20-sample group. dbcan-nf-CA, even though it detected more CAZyme families, had the highest computational cost (∼61 h and 132 GB RAM for the 20-sample group) and the worst scalability (bottom, Fig, 3B, Table 11, available as supplementary data at *Bioinformatics Advances* online). This is expected because coassembly of pooled reads from all 20 samples still ran on a single node on the HPC cluster and did not benefit from the HPC scheduling and distribution of multiple jobs to run in parallel.

Overall, dbcan-nf-IA, dbcan-nf-AF, dbcan-nf-SS, and Cayman all benefited from concurrent processing of multiple samples on an HPC system, whereas dbcan-nf-CA did not, as the coassembly of all read samples had to use a single node (bottom, Fig. 3B, Table 11, available as supplementary data at *Bioinformatics Advances* online).

### 3.4 dbcan-nf-IA works for long read samples

*Mock-stag-70* was sequenced as a single sample including 4.2 million Oxford Nanopore long reads (ONRs) with an average length of 2,645bp (Table 8, available as supplementary data at *Bioinformatics Advances* online) (Treichel *et al*. 2026). The sequencing reads were from 70 reference genomes with their CAZymes annotated by dbCAN as the ground-truth data (Table 1). Specifically, DNAs were individually extracted from 70 gut bacterial isolates and mixed in a staggered distribution (i.e. different relative abundances of different bacteria; Table 5, available as supplementary data at *Bioinformatics Advances* online) (Treichel *et al.* 2026). We randomly subsampled 0.5, 1, and 3 million reads, together with the full sample, corresponding to four different sample sizes (Table 8, available as supplementary data at *Bioinformatics Advances* online), to form the *Mock-stag-70 dataset*. As the read mapping-based Cayman and dbcan-nf-AF do not support long reads as input, we only included dbcan-nf-IA in this experiment to evaluate its CAZyme annotation accuracy (Fig. 4A) and scalability (Fig. 4B) when increasing the size of the single sample of long reads. This experiment was run on a standalone desktop Linux server with 32 CPUs and 200 GB of RAM.

**Figure 4:**
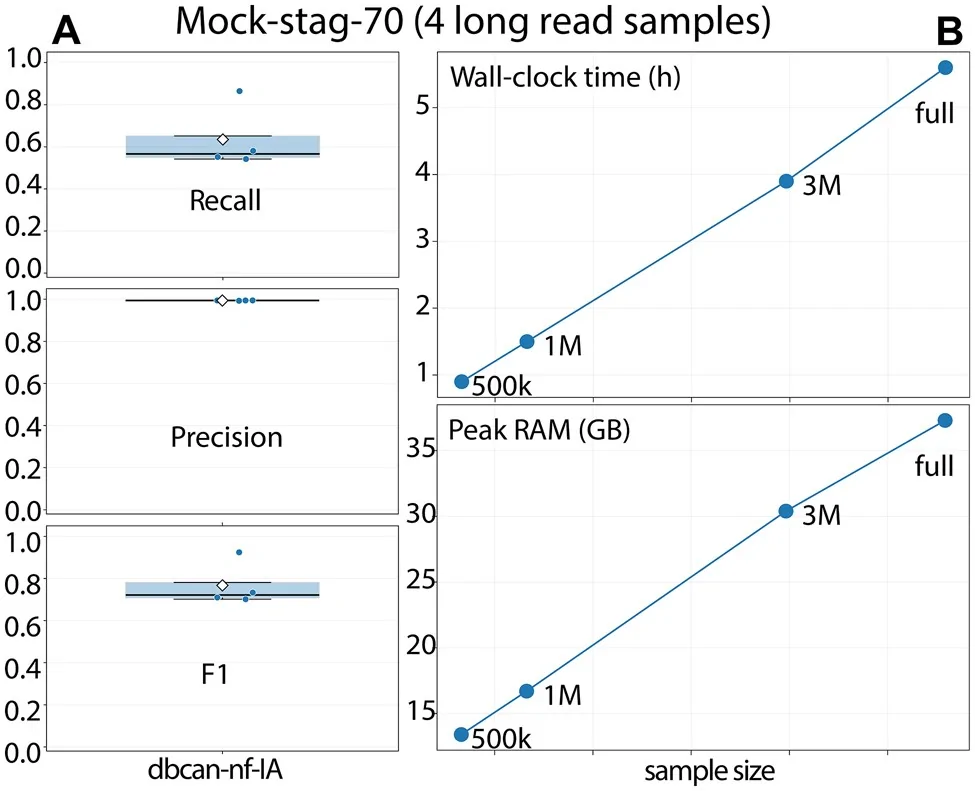
CAZyme annotation accuracy and computational cost of dbcan-nf-IA on Nanopore long read samples. (A) Boxplots of recall, precision, and F1 score of the four sequencing-depth subsets of Mock-stag-70. (B) Plots of sequencing depths (*x*-axis) versus Wall-clock times (*y*-axis) on the left, and sequencing depths in numbers of reads (*x*-axis) versus peak memory uses (*y*-axis) on the right.

We found dbcan-nf-IA had a 0.86 recall, 0.99 precision, and 0.92 F1 score when the full sample (4.2 million reads, 11.17 Gbp, Table 8, available as supplementary data at *Bioinformatics Advances* online) was used (Fig. 4A). However, the recall dropped to 0.58 in the subsample with 3 million reads (7.94 Gbp) and to 0.54 in the subsample with 0.5 million reads (1.32 Gbp), while the precision was consistently higher for smaller subsamples (>0.99). As a result, the mean F1 score for the four sequencing-depth subsets was 0.77. Clearly, sequencing depth is a key factor in determining the CAZyme annotation accuracy for the ONR dataset. This is likely because a sufficiently high read depth is required to correct the high error rate in the Oxford Nanopore long reads during the read assembly. As expected, with the increasing sample size, the assembly-based dbcan-nf-IA had a higher computing time and peak RAM use (Fig. 4B). The increasing wall-clock time and memory use observed with larger long-read inputs reflect the computational requirements of processing a single sample. In contrast, the workflow-level benefit of Nextflow is primarily realized when independent samples can be distributed and processed concurrently on an HPC system.

## 4 Discussion

Software tools taking WGS reads for CAZyme annotation have recently become available. We published a protocol with a step-by-step workflow (Zheng *et al.* 2024). However, it lacks robustness, scalability, and accessibility, because users have to install and run a collection of Bash one-liners and Python scripts in a manual fashion. Here, we implement the protocol as dbcan-nf by adopting the standardized and scalable Nextflow framework (Di Tommaso *et al.* 2017). dbcan-nf provides four different routes of CAZyme profiling in metagenomes for users to choose, covering both read mapping-based and assembly-based strategies. From the user’s perspective, it is important to know how to choose between these different routes. Cayman was recently published (Ducarmon *et al.* 2026) as a Python package for CAZyme profiling in WGS metagenomes. Cayman maps reads to a collection of precomputed habitat-specific reference gene catalogs (including CAZymes) from the GMGC (Coelho *et al.* 2022). With dbcan-nf, users would be interested in knowing the weaknesses and strengths of these available tools, and having answers to questions like, which tool is more accurate, which tool runs faster, and which tool is more scalable (e.g. when they have a large number of samples)? Here, we provided answers to these questions using multiple benchmark datasets.

Benchmarking with datasets containing ground-truth CAZyme annotations showed that dbcan-nf, especially dbcan-nf-IA is more accurate. In Fig. 2 and Table 1, we showed evidence that Cayman has much lower recall and F1 score than dbcan-nf-IA and dbcan-nf-AF on both CAZy-rand-10 and Mock-stag-24 datasets. In Fig. 2B and Table 7, available as supplementary data at *Bioinformatics Advances* online, we further showed evidence that dbcan-nf-IA was the more accurate method with an F1 score = 0.95 (recall = 0.91 and precision = 0.99) when the sequencing depth was 2.13 Gb. At the same sequencing depth 2.13 Gb, Cayman had an F1 score = 0.76 (recall = 0.62 and precision = 0.97), and dbcan-nf-IA-v9 had an F1 score = 0.93 (recall = 0.87 and precision = 0.99). On the Oxford Nanopore long read sample Mock-stag-70-full (Fig. 4 and Table 8, available as supplementary data at *Bioinformatics Advances* online), dbcan-nf-IA had an F1 score = 0.92 (recall = 0.86 and precision = 0.99). Cayman does not take long reads, so it was not possible to evaluate its accuracy. We think there are three possible reasons for the low recall of Cayman: (i) Cayman uses the rapid but less sensitive BWA-MEM for read mapping; (ii) Cayman uses a biased reference gene catalog lacking representation from understudied microbiomes (e.g. non-industrialized humans); and (iii) Cayman builds their precomputed reference CAZymes using an older version (v9) of dbCAN HMMdb (however, we showed dbcan-nf-IA-v9 and dbcan-nf-AF-v9 also performed better than Cayman).

Cayman runs faster and requires fewer computational resources than dbcan-nf. In Fig. 3B, Tables 9 and 11, available as supplementary data at *Bioinformatics Advances* online, we showed evidence that Cayman finished the jobs faster and used less RAM than all dbcan-nf routes on both Hadza-gut (except for Dry2014) and Wastewater datasets. We think the reason is primarily because Cayman uses the rapid BWA-MEM for read mapping against the smaller habitat-specific reference CAZyme gene catalogs. This computational efficiency is consistent with its use of BWA-MEM and smaller habitat-specific reference catalogs, although these choices also contribute to a lower recall.

For multisample analyses, dbcan-nf-IA, dbcan-nf-AF, dbcan-nf-SS, and Cayman could all benefit from running jobs concurrently through the Slurm scheduler on the HPC system. In Fig. 3B, we showed that all dbcan-nf routes (except for dbcan-nf-CA) and Cayman showed only modest increases in elapsed wall-clock time, while the maximum task-level RAM remained largely stable. For coassembly of more samples, dbcan-nf-CA could not benefit from HPC’s advantage for distribution of multiple jobs to run in parallel. The same was also true in Fig. 4B, where increasing the sample size (more reads) for assembly required more computing time and RAM.

In terms of how to choose from different dbcan-nf routes, our recommendation is that dbcan-nf-IA offers the best balance between accuracy and computational cost, and therefore it is made as the default route in dbcan-nf. When users have a small number of samples (e.g. 10) each with a moderate sequencing depth (e.g. >1.0 and <5 Gb), dbcan-nf-IA could finish within 1 h on an HPC cluster with a very good accuracy. If users choose Cayman or dbcan-nf-AF, they could finish in a shorter time but have the risk of losing a significant number of CAZymes (Fig. 3). This risk is much higher for Cayman when users have metagenomes from understudied human populations, hosts, and ecological environments. When the samples are sequenced at a very shallow depth (e.g. <0.5 Gb) or the microbiome has very high species diversity, the coassembly route (dbcan-nf-CA) is recommended because it could increase the depth by pooling reads from all samples (yes, these samples cannot be unrelated) to recover more low-abundance contigs. dbcan-nf-AF is a better alternative to Cayman as it is also fast but has much higher recall and F1 score. dbcan-nf-SS is generally not recommended as it tends to significantly underestimate CAZyme diversity (Figs 1D and 3B) and has no advantage over Cayman in terms of computational cost.

An additional practical advantage of dbcan-nf is its integration with the actively maintained dbCAN databases and software ecosystem (especially dbCAN HMMdb and CAZyme FASTAdb), and support of CAZyme annotation has been reliable, consistent, and continuous since 2012. For this reason, many metagenome data analysis software tools [including Cayman (Ducarmon *et al.* 2026)] have integrated data or tools from dbCAN into their development, such as the KBase (Chivian *et al.* 2023) and the metagenome annotation tool mettannotator (Gurbich *et al.* 2025).

Limitations of this study include (i) CAZyme abundance and substrate abundance are not considered and evaluated against benchmark datasets, and (ii) evaluation of PacBio long reads is not provided. We will conduct these analyses in our future work.

## Supplementary Material

vbag304_Supplementary_Data

## Data Availability

Source code of dbcan-nf is available at https://github.com/bcb-unl/dbcan-nf, and documentation at https://run-dbcan.readthedocs.io/en/latest/nextflow/index.html. The dbcan-nf workflow is built upon a previously developed stepwise protocol available at: https://run-dbcan.readthedocs.io/en/latest/metagenomics_pipeline/run_from_raw_reads.html.
