# Supplementary material for: dbcan-nf: a Nextflow pipeline for accurate CAZyme annotation in metagenomes
Source: Bioinform Adv. 2026 Oct 7;6(1):vbag304. doi: 10.1093/bioadv/vbag304 (PMC13679938; doi:10.1093/bioadv/vbag304)
Supplement: Supplementary file 1 [file vbag304_supplementary_data.zip › Supplement/dbcan-nf-SI-final.docx]

**Supplementary Methods**

**dbcan-nf: a Nextflow pipeline for accurate CAZyme annotation in metagenomes**

Xinpeng Zhang^1,*^, Haidong Yi^2,*^, Jinfang Zheng^3^, Yanbin Yin^1,#^

^1^ *Nebraska Food for Health Center, Department of Food Science and Technology, University of Nebraska - Lincoln, Lincoln, NE 68588, USA*

*^2^ St. Jude Children's Research Hospital, Memphis, TN 38105, USA*

*^3^ Chongqing Institute of Intelligent Medicine (CIIM), Chongqing, China*

** co-first authors*

*# corresponding author:* [*yyin@unl.edu*](mailto:yyin@unl.edu)

**1. Benchmark dataset collection**

We constructed five benchmark datasets (**Table S1**) to evaluate the performance of dbcan-nf and compare it against competing tools (**Table S2**). These datasets were used for different evaluation purposes, depending on how they were generated, the availability of ground-truth CAZyme annotation, the sequencing depth, and their sample sizes.

The *CAZy-rand-10 dataset* consists of simulated metagenomic reads from 10 RefSeq genomes (**Table S3**) with CAZy’s annotated CAZyme families (Drula et al. 2022), which are used as the ground-truth data for the calculation of recall, precision, and F1 score. Paired-end reads were simulated with CAMISIM (Fritz et al. 2019) to generate 10 random samples (**Table S6**) of the same size but with varying sequencing depths for the 10 RefSeq genomes (**Table S3**).

The *Mock-stag-24 dataset* and the *Mock-stag-70 dataset* are both taken from a recent publication (Treichel et al. 2026), which sequenced different mock communities with mixtures of DNA extracted from 24 and 70 cultured bacteria, respectively. All these bacteria have known reference genomes (**Table S4** for Mock-stag-24 and **Table S5** for Mock-stag-70), while their DNAs were mixed with staggered distribution (uneven amount) and sequenced at different depths to form different mock communities (Treichel et al. 2026). dbCAN (Zheng *et al.* 2023) was used to annotate these reference genomes to obtain CAZymes as the ground-truth data for the calculation of recall, precision, and F1 score.

We selected 18 samples sequenced by Illumina NovaSeq6000 (paired-end 150bp) with varying sequencing depths and of different sizes (**Table S7**) as the *Mock-stag-24 dataset.* The Oxford Nanopore long reads (**Table S8**) were sequenced by Promethion P2 solo with a total of 4.2 million reads. We subsampled 0.5, 1, and 3 million reads, together with the full sample, corresponding to four different sample sizes and forming the *Mock-stag-70 dataset*.

These three benchmark datasets allow a reference genome-based evaluation of CAZyme annotation recovery among different tools to assess CAZyme annotation accuracy, impact of sequencing depth, and route selection.

In addition, we also included two real-world metagenomic datasets: *Hadza-gut* (Carter *et al.* 2023) and *Wastewater* (Singh *et al.* 2026) to evaluate the scalability, computational cost, and route selection of dbcan-nf. The Hadza-gut dataset (**Table S9**) contains two samples from an ultra-deep sequencing study involving 351 fecal microbiome samples (Illumina paired-end 2x140bp reads) collected from Hadza hunter-gatherers in East Africa. The Wastewater dataset (**Table S10**) was included as a non-human-gut environmental microbiome benchmark, allowing us to evaluate dbcan-nf and Cayman in an understudied microbiome context. A total 20 samples (Illumina paired-end 2x150bp reads) were selected, and grouped into 5, 10, and 15 samples per group as the input to different routes of dbcan-nf and Cayman to evaluate the computational cost and scalability.

As these two datasets do not have reference genomes and ground-truth CAZymes, comparisons against other tools for recall, precision, and F1 score values are not possible. They were mainly used to evaluate the scalability and route selection of dbcan-nf.

The read sample information of all five benchmark datasets is provided in **Tables S6 to S10**, such as SRA accessions, read length, and read counts.

**2. Comparative evaluation of CAZyme annotation**

Three datasets (*CAZy-rand-10*, *Mock-stag-24,* and *Mock-stag-70*) have reference genomes and their genome-based CAZyme annotations were used as the ground-truth data to calculate the recall, precision, and F1 values for the performance comparison between different tools (**Tables S6,7,8**). *Mock-stag-24* has 18 samples with increasing amounts of input reads, and therefore is also used to evaluate the impact of sequencing depth on the CAZyme annotation accuracy of different tools.

These tools include dbcan-nf and Cayman (Ducarmon *et al.* 2026), which can profile CAZymes with metagenomic reads as the input. We also included run_dbcan, CUPP (Barrett *et al.* 2023), CAZyLingua (Thurimella *et al.* 2025), which do not directly accept metagenomic reads as input (**Table S2**). For these three tools, reads were first assembled into contigs using MEGAHIT (Li *et al.* 2015). Protein-coding genes were then predicted from the assembled contigs using Pyrodigal (Larralde 2022), generating protein FASTA files for downstream annotation. The predicted protein sequences were used directly as input for run_dbCAN and CUPP. For CAZyLingua, protein sequences were first converted into protein embeddings using ProtT5 (Elnaggar *et al.* 2022), and these embeddings were then used for CAZyLingua-based CAZyme prediction. The embedding step was included in calculating the full workflow runtime for CAZyLingua.

For dbcan-nf and Cayman, metagenomic reads in FASTQ format were used directly as input. dbcan-nf was run using the individual sample assembly (IA) route and the assembly free route (AF). Cayman does not assemble reads but instead maps reads to a precomputed reference CAZyme gene catalogs grouped according to different ecological environments. Therefore, Cayman was run with reads in FASTQ format as the input, and specifying the corresponding reference gene catalogs (**Table S12**), e.g., the human gut reference CAZyme gene catalog for *CAZy-rand-10* and *Hadza-gut*, the combined human and mouse gut reference CAZyme gene catalog for *Mock-stag-24*, and the wastewater reference CAZyme gene catalog for *Wastewater*. This allowed us to evaluate Cayman under catalog settings matched to the corresponding microbiome context.

For the two real-world metagenomic datasets: *Hadza-gut* and *Wastewater*, we do not have reference genomes and their CAZymes as the ground truth data. Therefore, evaluation of CAZyme annotation performance (e.g., recall, precision, and F1 score values) was not possible. In addition, Cayman has small, precomputed reference CAZyme gene catalogs for microbiomes from wastewater, freshwater, marine, other aquatic environments, and soil. Some of the most studied microbiomes from other animal hosts and ecological environments are excluded from Cayman’s CAZyme gene catalogs, such as cow rumens, plants, and hydrothermal vents. CAZyme gene catalogs for the human gut microbiome also lack representation from less studied human populations (e.g., Africans and South Americans). Therefore, the *Hadza-gut* and *Wastewater* benchmark datasets were selected to run dbcan-nf and Cayman to compare their annotated CAZyme families and glycan substrate profiles.

For *Hadza-gut*, we recorded the wall-clock runtime, peak RAM, and disk usage for the four routes of dbcan-nf and Cayman, as well as the number of predicted CAZymes, families, CGCs, and substrates (**Table S9**).

For *Wastewater*, we also compared the scalability of the four routes of dbcan-nf and Cayman by comparing the wall-clock runtime, peak RAM, and disk usage of different numbers of input samples (**Table S11**).

For all benchmarks, the same computing allocation of 32 CPUs and 200 GB of RAM was used unless otherwise specified. Software versions, database versions, command-line parameters, dataset-specific settings, and detailed resource usage statistics are provided in **Table S12**.

**3. Performance metrics**

Three benchmark datasets have reference genomes, and their genome-based CAZyme annotations were used as the ground-truth data (**Tables S6,7,8**). Once simulated (*CAZy-rand-10*) or sequenced reads (*Mock-stag-24,* and *Mock-stag-70*) were processed by different tools, true positives (TP) were defined as CAZyme families present in both predictions and ground-truths. False positives (FP) were defined as predicted CAZyme families absent from ground-truths. False negatives (FN) were defined as CAZyme families in ground-truths but absent in predictions. Precision, recall, and F1 score were calculated at the CAZyme family level:

Recall=TP/((TP+FN))

Precision=TP/((TP+FP))

F1=2×(Precision × Recall)/((Precision+Recall))

**Reference**

Barrett K, Hunt CJ, Lange L *et al.* Conserved unique peptide patterns (CUPP) online platform 2.0: implementation of +1000 JGI fungal genomes. *Nucleic Acids Res* 2023;**51**(W1):W108–14. https://doi.org/10.1093/nar/gkad385.

Carter MM, Olm MR, Merrill BD *et al.* Ultra-deep sequencing of Hadza hunter-gatherers recovers vanishing gut microbes. *Cell* 2023;**186**(14):3111-3124.e13. https://doi.org/10.1016/j.cell.2023.05.046.

Drula E, Garron ML, Dogan S *et al.* The carbohydrate-active enzyme database: functions and literature. *Nucleic Acids Research* 2022;**50**(D1):D571–7. https://doi.org/10.1093/nar/gkab1045.

Ducarmon QR, Karcher N, Giri S *et al.* Cayman enables large-scale analysis of gut microbiome carbohydrate-active enzyme repertoires. *Nat Microbiol* 24 Apr. 2026:1–15. https://doi.org/10.1038/s41564-026-02318-2.

Elnaggar A, Heinzinger M, Dallago C *et al.* ProtTrans: Toward Understanding the Language of Life Through Self-Supervised Learning. *IEEE Transactions on Pattern Analysis and Machine Intelligence* 2022;**44**(10):7112–27. https://doi.org/10.1109/TPAMI.2021.3095381.

Fritz A, Hofmann P, Majda S *et al.* CAMISIM: simulating metagenomes and microbial communities. *Microbiome* 2019;**7**(1):17. https://doi.org/10.1186/s40168-019-0633-6.

Larralde M. Pyrodigal: Python bindings and interface to Prodigal, an efficient method for gene prediction in prokaryotes. *Journal of Open Source Software* 2022;**7**(72):4296. https://doi.org/10.21105/joss.04296.

Li D, Liu CM, Luo R *et al.* MEGAHIT: an ultra-fast single-node solution for large and complex metagenomics assembly via succinct de Bruijn graph. *Bioinformatics* 2015;**31**(10):1674–6. https://doi.org/10.1093/bioinformatics/btv033.

Singh NK, Garg P, Kumari S *et al.* Metagenomic profiling of antimicrobial resistance in wastewater from metropolitan cities of India. *Nat Commun* 2026;**17**(1):4097. https://doi.org/10.1038/s41467-026-70702-x.

Thurimella K, Mohamed AMT, Li C *et al.* Protein language models uncover carbohydrate-active enzyme function in metagenomics. *BMC Bioinformatics* 2025;**26**(1):285. https://doi.org/10.1186/s12859-025-06286-y.

Treichel NS, Pauvert C, Séneca J *et al.* Benchmarking of shotgun sequencing depth reveals the potential and limitations of shallow metagenomics and strain-level analysis. *Nat Microbiol* 2026;**11**(5):1233–44. https://doi.org/10.1038/s41564-026-02334-2.

Zheng J, Ge Q, Yan Y *et al.* dbCAN3: automated carbohydrate-active enzyme and substrate annotation. *Nucleic Acids Research* 2023;**51**(W1):W115–21. https://doi.org/10.1093/nar/gkad328.
